# Child and Adolescent Social Adaptive Functioning Scale: Factorial Invariance, Latent Mean Differences, and Its Impact on School Refusal Behavior in Spanish Children

**DOI:** 10.3389/fpsyg.2019.01894

**Published:** 2019-08-14

**Authors:** Carolina Gonzálvez, Cándido J. Inglés, Ainhoa Martínez-Palau, Ricardo Sanmartín, María Vicent, José M. García-Fernández

**Affiliations:** ^1^Department of Developmental Psychology and Didactics, University of Alicante, Alicante, Spain; ^2^Department of Health Psychology, Miguel Hernandez University of Elche, Elche, Spain

**Keywords:** social functioning, validation, factorial invariance, latent mean differences, school refusal behavior, primary education, Spain

## Abstract

This study aims to examine the factorial invariance and latent mean differences across gender of the Spanish version of the Child and Adolescent Social Adaptive Functioning Scale (Study 1) and to value the function of social functioning as a protective ability of school refusal behavior (Study 2). Participants were Spanish students aged 8–12 years carefully chosen by simple random cluster, 345 for the first study (*M* = 9.17; SD = 1.03) and 1,032 students for the second study (*M* = 10.02; SD = 1.77). The measures used were the Child and Adolescent Social Adaptive Functioning Scale (CASAFS) and the School Refusal Assessment Scale-Revised (SRAS-R). Results about the validation of the scale supported the model proposed in this study for the CASAFS, with 15 items and a four-factor structure (school performance, peer relationships, family relationships, and home duties/self-care). Findings revealed invariance across gender for this model and good internal consistency levels were exhibited in each of the four dimensions of the CASAFS (0.76, 0.72, 0.74, and 0.71). Latent mean differences did not report differences between boys and girls. Regarding the second study, the social functioning acted as a protective factor of school refusal behavior by negatively and significantly predicting high scores in school refusal behavior due to anxiety symptoms or feelings of negative affect linked to the obligation to attend school. Opposite results were found for those students who justify their refusal to attend school in pursuing tangible reinforcements outside the school setting. These findings strengthen the reliability and validity of the CASAFS and the idea of social functioning as a person’s ability which could prevent school refusal behavior is discussed.

## Introduction

Social functioning is understood as a set of different dimensions, known as social cognition, social skills and interactions, and social behaviors (Beauchamp and Anderson, 2010). Therefore, social functioning refers to a wide construct encompassing cognitive, emotional, and linguistic skills (Crowe et al., 2011). Social functioning difficulties are related with different sorts of psychological disorders, both internalizing problems such as depression (Vuthiarpa et al., 2012) or anxiety (Alfano, 2012; Essau et al., 2012), and externalizing problems such as conduct disorders (Renouf et al., 1997). Therefore, it is necessary to be able of using social functioning measures to identify social and adaptive functioning deficits in the early stages of human development. However, the development of measures evaluating social functioning through precise indicators has received scant attention. In this regard, the Child and Adolescent Social and Adaptive Functioning Scale (CASAFS; Spence et al., 2000) is a self-report measure specifically developed to examine the social and adaptive functioning of young people in the areas of school performance, peer relationship, family relationship, and home duties/self-care. Price et al. (2002) examined its psychometric properties in 1,478 Australian adolescents (*M* = 12.85; SD = 0.54). The results reported good internal consistency (*α* = 0.81) and moderate test-retest reliability (*r* = 0.58) of the CASAFS. In this study, girls obtained significantly higher scores than boys on the peer relationships and the home duties/self-care subscales. Despite these findings, no further investigations have proved its psychometric properties in other cultures.

In recent years, social functioning has become more relevant due to its influence on social competence (McQuade et al., 2013), as well as on other variables related to the academic field such as school performance (Gutiérrez et al., 2011). A large number of investigations reveal a positive relationship between social-emotional competence and academic success (Miller et al., 2005; Talwar et al., 2017; Vicent et al., 2017). At the same time, there is empirical evidence to suggest that antisocial behavior is a statistically significant and positive predictor of school failure (Raine et al., 2006). This is why social functioning is considered as an important variable involved in school adaptation (Duncan et al., 2007; Furguerle and Graterol, 2010; Fernández-Zabala et al., 2016).

Despite the existing works about social functioning as a variable that facilitates an adequate socio-emotional adjustment, the theoretical revision shows the scarcity of works that have analyzed its role as a protective factor of school problems that affect the current society. School refusal behavior is included among the mentioned issues and it is referred to a child’s refusal to go to school regularly or the persistent difficulty of staying in school (Kearney, 2016). In the last few years, most of the studies that have analyzed the relationship between school refusal and different personality traits and emotional states have chosen variables that fundamentally have a negative impact on school attendance problems. The findings of these studies reveal that school refusal is associated with higher scores in anxiety (Kearney and Albano, 2004; Gonzálvez et al., 2018a), depression (Egger et al., 2003; Gonzálvez et al., 2018b) or pessimism (Gonzálvez et al., 2018c). However, this work pretends to analyze the impact of a variable, social functioning, whose high levels are expected to have a positive effect and lead to lower school refusal rates.

Few previous studies have considered the relationship between school refusal behavior and social functioning understood as a multidimensional construct that includes school performance, home duties/self-care, and the relationship with family and friends. In fact, just one has been recently found in which the relationship between four different school refusal behavior profiles and social functioning was analyzed (Gonzálvez et al., 2019). In this study, the non-school refusers group achieved the highest average scores in social functioning. On the other hand, other variables related to social functioning (e.g., social skills) have been analyzed in the field of school attendance problems. Specifically, Egger et al. (2003) noted that students who refuse to attend school or present anxiety disorders often have poor interpersonal relationships.

The consequences of not attending school on a long-term basis generate a decrease in the levels of social functioning (Havik et al., 2015). For this reason, individuals presenting school attendance problems are more prone to present problems during social situations, particularly when making and keeping friends (Wilson et al., 2008; Carroll, 2011; Gonzálvez et al., 2016). In this sense, there are studies that suggest that having good friends can prevent the appearance of school refusal (Shilvock, 2010; Havik et al., 2014). In addition, they prove that the difficulty of attending school could be caused by showing poor stability in different social situations (Havik et al., 2015).

Despite these gains, no previous studies have presented the Spanish validated version of the CASAFS or have analyzed the predictive capability of social functioning on school refusal behavior. These two limitations intend to be solved by this work. On the one hand, it will offer for the first time the validation of the CASAFS in a different cultural environment, specifically Spanish children. On the other hand, it will check the influence of social functioning on the manifestation of school non-attendance problems. Therefore, the purpose of the present study is twofold. First, it attempts to test the factorial invariance of the Spanish version of the CASAFS with Spanish children from third to six grade of Primary Education. Second, it purports to value the function of social functioning as a protective ability of school refusal behavior. In particular, this study aims (1) to check if it replicates the four-factor structure of the CASAFS, (2) to test its reliability, (3) to determine the factorial invariance of the CASAFS across gender, (4) to analyze the latent mean differences across gender, and (5) to determine the relationship between social functioning and school refusal behavior.

Taking into account the findings reported by previous studies, it is expected that:

Hypothesis 1. The Spanish version of the CASAFS presents the four-dimensional structure (Spence et al., 2000; Price et al., 2002).
Hypothesis 2. The Spanish version of the CASAFS obtains adequate coefficients of internal consistency in accordance with the previous studies (Spence et al., 2000; Price et al., 2002).
Hypothesis 3. It remains invariant across gender.
Hypothesis 4. It reports differences across gender with girls achieving higher social functioning scores than boys (Price et al., 2002).
Hypothesis 5. Students with high social functioning scores obtain the lowest scores in school refusal behavior (Duncan et al., 2007; Furguerle and Graterol, 2010; Fernández-Zabala et al., 2016).
Hypothesis 6. Social functioning acts as a statistically significant predictor in a negative sense of high scores in school refusal behavior (Gonzálvez et al., 2019).

## Study 1

Validation, factorial invariance, and latent mean differences across gender for the CASAFS in Spanish children population.

### Method

#### Participants

At the beginning, this study included 397 children recruited by random cluster sampling in the province of Alicante (geographical areas: center, north, south, east, and west). In the selection process, six urban and rural schools were chosen. Some of the initial sample participants were removed because they did not deliver the paternal consent to collaborate in the research (*N* = 24), they had omissions when completing the questionnaires (*N* = 21) and they had an insufficient language proficiency to understand the Spanish (*N* = 17). Thus, the final sample comprised a normative sample of 345 Spanish children aged 8–12 years (*M* = 9.17; SD = 1.03), of which 43.8% were boys and 56.2% were girls. Uniform distribution across gender and age was revealed according to the *χ*^2^ test (*χ*^2^ = 7.04, *p* = 0.07).

Childhood socioeconomic status was determined according to parental occupation (employed or unemployed) and education background (primary, secondary or higher education). The sample included families with different socioeconomic status but with a predominance of middle-class children (67% employed families and 21% primary education; 48% secondary education; 31% higher education).

#### Measure

Child and Adolescent Social Adaptive Functioning Scale (CASAFS; Spence et al., 2000). The CASAFS is a self-report measure that assesses social functioning in children and adolescents understood as the degree to which an individual fulfills various roles in his or her life. This scale is composed by 24 items distributed in four subscales: School Performance (SP; e.g., “*I get good marks in social science and/or history*”), Peer Relationships (PR; e.g., “*I have at least one or two special friends*”), Family Relationships (FR; e.g., “*I get on well with my relatives*”), and Home Duties/Self-care (HD; e.g., “*I help with the cleaning up after meals*”). Items are scored on a four-point Likert-type scale (1 = Never; 4 = Always). Family relationship items included a fifth scoring category stating “*does not apply to me*” in case of those individuals without siblings or one of their parents. The instrument has shown adequate levels of internal consistency (0.67–0.81) and a test-retest reliability with a 12-month interval of 0.48–0.63 (Price et al., 2002). Construct validity of this measure was supported by a negative and significant correlation found between the total CASAFS scores and total scores on the Beck Depression Inventory (BDI; Beck et al., 1961; Price et al., 2002).

The back-translation method was used to translate this scale to Spanish. First, the CASAFS items were translated from English to Spanish by a translator who was a native speaker with knowledge in the field and university studies in English translation. Then, the Spanish version of the CASAFS was translated back into the source language by an independent translator who was a native English speaker with Spanish knowledge and studies in Psychology. Finally, the two source-language versions were then compared.

#### Procedure

First, an interview was conducted with the principals of the centers explaining the aims of the investigation and describing the evaluation instrument. Once they accepted their participation, legal custodians were asked for the written informed consent. After collecting the authorizations during 2 weeks, students anonymously and collectively completed the instrument in a 20-min session during school hours, at least one of the researchers was always present to solve doubts. Once the instrument was applied, all the groups (students, families, teachers, and principals) were thanked for participating and the research group undertook to send a report with the results and orientations about educational support. Besides, the study followed the ethics standards established by the Declaration of Helsinki and the research study protocol was approved by the Ethical Committee of the University of Alicante (UA-2017-09-05).

#### Statistical Analyses

A Confirmatory Factorial Analysis (CFA) was carried out to test the dimensional structure of the CASAFS and consider its adequacy in Spanish children. The robust Maximum Likelihood method was used. No multivariate normality was identified according to Mardia’s coefficient (17.57) (Bentler, 2005) and as a consequence, the Satorra-Bentler scaled *χ*^2^ (S-B*χ^2^*) was used. Four goodness-of-fit indexes were considered: the Robust Root Mean Square Error of Approximation (R-RMSEA) with scores lower than 0.08 considered acceptable and lower than 0.06 excellent; the Robust Comparative Fit Index (R-CFI) with scores equal or greater than 0.90 considered acceptable and larger than 0.95 good fit; the Standardized Root Mean Square Residual (SRMR) with scores close to 0.08 considered acceptable and lower than 0.05 good fit; and the Tucker Lewis Index (TLI) with scores equal or greater than 0.90 considered acceptable (Hu and Bentler, 1999; Brown, 2006). In addition, the internal consistency of the CASAFS was obtained through Cronbach’s alpha coefficient and a classic item analysis was carried out.

Second, the configural, measurement and structural invariance of the own model of the CASAFS across gender was performed by Multigroup Confirmatory Factorial Analysis (MGCFA). In accordance with the scores obtained in the Mardia’s coefficient (>5), the S-B*χ*^2^ was consequently used. Several hierarchical steps were followed and the goodness-of-fit indexes were calculated along with the following invariance criteria: the adjusted Satorra-Bentler Chi-square difference (ΔS-B*χ*^2^: *p* > 0.05) and the ΔCFI (ΔCFI < 0.01). The latent mean differences across gender were performed with the Critical Ratio statistic (CR). Statistical analyses were calculated using the IBM SPSS Statistics Base 22.0 and the multivariate software EQS 6.1.

### Results

#### Confirmatory Factor Analysis and Reliability

Table 1 presents the CFA results for the original model and the own model proposed by this research group. It was the own model which reported the best goodness-of-fit indexes, which were higher than 0.90 for R-CFI (0.989) and TLI (0.986) and with an excellent value for R-RMSEA (0.016) and SRMR (0.065). This model supports the four-factor structure of the CASAFS after removing nine items and establishing item correlations. As a result, a final structure formed by 15 items of the CASAFS is proposed: School Performance (SP): 1, 5, 9, 13, and 21; Peer Relationships (PR): 14, 18, and 22; Family Relationships (FR): 7, 15, and 19; Home Duties/Self-care (HD): 8, 12, 16, and 24.

**Table 1:** Confirmatory factor analyses: goodness-of-fit indexes of the statistic models of the CASAFS.

	S-B*χ*^2^	df	R-RMSEA 90% CI	SRMR	R-CFI	TLI
Original model	355.9596	246	0.051 (0.039, 0.062)	0.083	0.818	0.796
Own model	83.4619	80	0.016 (0.000, 0.046)	0.065	0.989	0.986

*Original model: Price et al. (2002); S-B*χ*^2^, Satorra-Bentler scaled *χ*^2^; df, degrees of freedom; R-RMSEA, robust root mean square error of approximation; CI, confidence interval; SRMR, standardized root mean square residual; R-CFI, robust comparative fit index; TLI, Tucker Lewis index*.

*p < 0.001 for S-B*χ*^2^ in all cases*.

Cronbach’s alpha values for each of the four factors were 0.76 (SP), 0.72 (PR), 0.74 (FR), and 0.71 (HD).

#### Classical Item Analysis

Item means ranged between 1.89 (item 5) and 2.76 (item 15) and the standard deviation ranged between 0.58 (item 15) and 1.04 (item 22). The item-test correlation coefficients ranged from 0.33 (item 14) to 0.57 (item 13). The items that did not reach a correlation coefficient of 0.30 were deleted. Thus, the CASAFS is composed by 15 items related to the functioning and social adaptation of children.

The items-subscales correlation coefficients ranged from 0.61 (item 9) to 0.76 (item 13) in the first factor (SP), from 0.65 (item 14) to 0.73 (item 22) in the second factor (PR), from 0.71 (item 19) to 0.76 (item 15) in the third factor (FR), and from 0.59 (item 16) to 0.80 (item 12) in the fourth factor (HD). The internal consistency (Cronbach’s alpha) of the questionnaire, if an item is removed, oscillates between 0.70 and 0.72.

#### Factorial Validity and Invariance Across Gender

Table 2 shows the measurement and structural invariance across gender by performing different multigroup analyses. The baseline model (Model 0), with no constraints, revealed adequate goodness-of-fit for the TLI, R-CFI, R-RMSEA, and SRMR indexes. The model 1, obtained after imposing constraints in the factor loadings of Model 0, revealed acceptable goodness-of-fit indexes also. Consecutively, the equality of intercepts was fixed in Model 1 and a new model was obtained (Model 2) with adequate goodness-of-fit indexes. The strict invariance, represented by the Model 3 obtained satisfactory goodness-of-fit indexes also concluding thus the measurement invariance. Finally, the structural invariance (Model 4), which constrains the variances and covariances of factors in Model 2, obtained satisfactory goodness-of-fit indexes. All the ΔS-B*χ^2^* of the different models showed no statistically significant differences (*p* > 0.05), and the ΔCFI values were lower than 0.01. These data confirm the measurement and structural invariance of the CASAFS across gender.

**Table 2:** Goodness-of-fit indexes for the own model of the CASAFS depending on gender.

	*χ*^2^	S-B*χ*^2^	df	TLI	R-CFI	R-RMSEA	SRMR	ΔS-B*χ*^2^ (Δdf, *p*)	ΔCFI
Boys	95.866	81.5334	80	0.989	0.991	0.016 (0.000, 0.068)	0.066		
Girls	118.894	91.7004	80	0.902	0.925	0.039 (0.000, 0.071)	0.077		
Model 0	214.773	175.1688	160	0.940	0.954	0.024 (0.000, 0.043)	0.072		
Model 1	227.068	182.8608	171	0.952	0.961	0.021 (0.000, 0.041)	0.072	8.36 (11, 0.680)	0.007
Model 2	232.496	190.3151	186	0.938	0.952	0.017 (0.000, 0.039)	0.072	5.48 (15, 0.987)	−0.009
Model 3	254.095	206.8305	205	0.949	0.959	0.014 (0.000, 0.036)	0.078	16.67 (19, 0.613)	0.007
Model 4	238.373	197.0030	196	0.954	0.961	0.014 (0.000, 0.036)	0.078	5.91 (10, 0.823)	0.009

*Model 0 = free model; Model 1 = Model 0 with factor loadings; Model 2 = Model 1 with intercepts; Model 3 = Model 2 with error variances; Model 4 = Model 2 with variances and covariance factors; S-B*χ*^2^, Satorra-Bentler *χ*^2^ scaled; df, degrees of freedom; TLI, Tucker-Lewis index; R-CFI, robust comparative fit index; R-RMSEA, robust root mean square error of approximation; SRMR, standardized root mean square residual; ΔCFI, comparative fit index difference test; ΔS-B*χ*^2^, *χ*^2^ difference model comparison test; Δdf, difference between degrees of freedom*.

#### Latent Mean Differences Across Gender

To compare the differences in social functioning across gender, boys acted as the gender reference group (see Table 3). Reasonable goodness-of-fit indexes were obtained for both groups across gender (*χ*^2^ = 267.016, d.f. = 182, *p* < 0.000, R-CFI = 0.929, R-RMSEA = 0.037, CI = 0.016–0.052, and SRMR = 0.077). Not statistically significant differences were found across gender in the CASAFS scores.

**Table 3:** Latent mean differences across gender in the CASAFS.

	CASAFS			
	SP	PR	FR	HD
Boys (reference)				
Girls				
Mean estimate (ME)	−0.008	−0.019	−0.016	0.131
Standard error (SE)	0.099	0.066	0.083	0.116
Critical ratio (CR)	−0.085	−0.287	−0.191	1.126

*SP, school performance; PR, peer relationship; FR, family relationship; HD, home duties/self-care*.

### Discussion

The aim of the first study was to carry out the validation of the CASAFS in a sample of Spanish children. As expected, the CFA supported the four-factor structure of the scale (Hypothesis 1), coinciding with the previous models (Spence et al., 2000; Price et al., 2002). Regarding the reliability, the second hypothesis was confirmed because the CASAFS reported adequate levels of reliability, which ranged from 0.76 (SP) to 0.71 (HD). In this sense, these values are considered to be acceptable because they are equal or greater than 0.70 according to Prieto and Delgado (2010). In addition, the MCFA confirmed the configural, measurement, and structural invariance of the proposed model by this research for the CASAFS across gender, so the third hypothesis was also accepted. With regard to the results about the latent mean differences across gender, initial expectations have not been met (Hypothesis 4). In this study, no significant differences were found between boys and girls in social functioning. Despite relatively few studies that have analyzed the differences across gender in social functioning, higher scores were associated to girls (Price et al., 2002; Bree, 2004). However, these findings might be justified by the fact that in previous studies these differences were examined with adolescents’ samples whereas in this investigation are children. Moreover, the school and family environment of the children of the current research could explain these results.

## Study 2

Social functioning as a protective factor of school refusal behavior: mean differences and predictive capability.

### Method

#### Participants

The sample was recruited by random cluster sampling in four Spanish cities: Alicante, Albacete, Murcia, and Seville. Five different geographical areas were considered (center, north, south, east, and west) in the selection process. Finally, 16 town and rural schools were chosen (11 public, 3 concerted, and 2 private schools), in which four classes per center were randomly selected and an average participation rate of 61 students per school was reached.

The final sample included a normative sample of 1,032 students, after excluding 62 contributions for presenting mistakes and omissions during the fulfillment of the tests or for not having the written consent of their legal tutors. Ages of these participants ranged from 8 to 12 years (*M* = 10.02; SD = 1.77). Uniform distribution across gender and age was revealed according to the *χ*^2^ test for uniform (*χ*^2^ = 3.04, *p* = 0.31).

The socioeconomic status was determined according to parental occupation (employed or unemployed) and education background (primary, secondary or higher education). The sample included families with different socioeconomic status but with a predominance of middle-class children (73% employed families and 26% primary education; 51% secondary education; 33% higher education).

#### Measures

Child and Adolescent Social Adaptive Functioning Scale (CASAFS; Spence et al., 2000). Its characteristics and psychometric properties have been explained before. In this study, the coefficients of internal consistency were 0.75 (SP), 0.70 (PR), 0.71 (FR), and 0.73 (HD) for each of the four factors, respectively.

School Refusal Assessment Scale-Revised (SRAS-R; Kearney, 2002). The SRAS-R is a self-report measure designed to identify the primary function that explains school refusal behavior through four dimensions: I. Avoidance of stimuli that provoke negative affectivity (e.g., “*How often do you stay away from school because you will feel sad or depressed if you go?*”), II. Escape from aversive social and/or evaluative situations (e.g., “*How often do you stay away from places in school (e.g., hallways, places where certain groups of people are) where you would have to talk to someone?*”), III. Pursuit of attention from significant others (e.g., “*How much would you rather be with your family than go to school?*”), and IV. Pursuit of tangible reinforcement outside of school (e.g., “*When you are not in school during the week (Monday to Friday), how often do you leave the house and do something fun?*”). The SRAS-R includes 24 items with a seven-point Likert-type scale (0 = Never; 6 = Always). Both the original and revised version have demonstrated adequate psychometric properties obtaining Cronbach alpha values for the SRAS-R that ranged from 0.74 (Factor IV) to 0.87 (Factor III) (Kearney, 2006).

In this study, the Spanish version of the SRAS-R was used with a structure of 18 items divided into the four factors mentioned above (Gonzálvez et al., 2016). Adequate coefficients of internal consistency were found with a range from 0.70 (Factor I) to 0.87 (Factor III). Correlation coefficients of scores of the SRAS-R revealed a predictable pattern between school refusal behavior and positive/negative affect and optimism/pessimism. Specifically, Gonzálvez et al. (2016) revealed positive and significant correlations between the first three factors and the total score of the SRAS-R with negative affect and pessimism. In this study, the coefficients of internal consistency were 0.77 (Factor I), 0.78 (Factor II), 0.73 (Factor III), and 0.71 (Factor IV) for each of the four dimensions, respectively.

#### Procedure

First, an interview with the principals of the centers was carried out and written informed consent was requested from the parents. Participants anonymously and collectively completed the instruments in a 45-min session during school hours (5 min presentation and detailed guidance to complete the instruments, 15–20 min the CASAFS, and 15–20 min the SRAS-R).

#### Statistical Analyses

The Student’s *t* test was used to examine the differences in the mean scores of students with high and low school refusal behavior depending on the social functioning. In accordance with the values proposed by Cohen (1988) to interpret the magnitude of the effect sizes, three levels were differentiated: small (0.20 < *d* < 0.50), moderate (0.51 < *d* <. 0.79), and large (*d* ≥ 0.80).

Binary logistic regression process was used to analyze the predictive capability of the social functioning on high scores in school refusal behavior. The OR statistic based on Wald’s statistic was used to interpret the results: scores greater than one showed a positive prediction, scores smaller than one indicated negative predictions, and scores equal to one showed no prediction. In this case, only the IBM SPSS Statistics Base 22.0 was used.

### Results

#### Mean Differences

Differentiating between students with high and low school refusal behavior scores, Table 4 presents the mean scores of these two groups across social functioning. Students with low scores in the first three factors of the SRAS-R obtained higher scores in three dimensions of social functioning (school performance, peer relationships, and family relationships) than their peers with high scores. The magnitude of the differences found were small and moderate, ranging between 0.22 and 0.73.

**Table 4:** Differences in social functioning in students with high and low scores in school refusal.

Variables		Levene’s test		Low score		High score		Statistics			
		*F*	*p*	*M*	SD	*M*	SD	*t*	df	*p*	*d*
SP	I SRAS-R	0.69	0.405	15.31	2.88	13.16	3.04	9.97	760	<0.001	0.73
	II SRAS-R	21.87	0.000	15.09	2.64	14.05	3.22	9.30	663.89	<0.001	0.35
	III SRAS-R	0.41	0.519	15.25	2.96	14.00	3.09	5.09	628	<0.001	0.41
	IV SRAS-R	4.41	0.036	12.92	2.63	15.25	2.80	−9.28	290.04	<0.001	0.85
PR	I SRAS-R	0.44	0.504	15.29	3.18	14.21	2.95	4.87	760	<0.001	0.35
	II SRAS-R	9.82	0.002	15.83	2.73	14.05	3.22	7.86	683.80	<0.001	0.59
	III SRAS-R	0.09	0.753	15.56	3.11	14.63	3.08	3.71	628	<0.001	0.30
	IV SRAS-R	0.52	0.468	13.42	2.55	16.11	2.91	−10.18	586	<0.001	0.95
FR	I SRAS-R	18.69	0.000	16.24	2.52	14.57	2.95	8.39	755.50	<0.001	0.61
	II SRAS-R	25.80	0.000	16.18	2.47	14.21	3.25	8.97	666.10	<0.001	0.68
	III SRAS-R	0.11	0.744	15.81	2.92	15.18	2.89	2.71	628	<0.001	0.22
	IV SRAS-R	28.85	0.000	15.00	2.83	16.31	2.20	−5.26	226.18	<0.001	0.55
HD	I SRAS-R	1.14	0.286	14.44	4.49	13.90	4.24	1.68	760	0.092	–
	II SRAS-R	2.05	0.152	14.70	4.52	13.90	4.17	2.44	688	0.015	0.18
	III SRAS-R	0.14	0.702	14.31	4.45	14.27	4.60	0.10	628	0.914	–
	IV SRAS-R	1.51	0.220	13.84	4.33	15.38	3.68	−4.26	586	<0.001	0.40

*SP, school performance; PR, peer relationships; FR, family relationships; HD, home duties/self-care; SRAS-R, school refusal assessment scale-revised; I SRAS-R, avoidance of stimuli that provoke negative affectivity; II SRAS-R, escape from aversive social and/or evaluative situations; III SRAS-R, pursuit of attention from significant others; IV SRAS-R, pursuit of tangible reinforcement outside of school*.

In contrast to these findings, students with high scores in school refusal behavior for the fourth factor (IV. tangible reinforcements) achieved higher scores in the four dimensions of social functioning than their companions with low scores, and the size of the differences found was large for the subscales school performance and peer relationships (*d* = 0.85, *d* = 0.95, respectively), moderate for family relationships (*d* = 0.55), and small for home duties/self-care (*d* = 0.40).

#### Predictive Capability

Logistic regression results are presented in Table 5. The percentage of cases correctly classified ranged from 63% (*χ*^2^ = 30.58; *p* = <0.001) for the third factor of the SRAS-R to 78.6% (*χ*^2^ = 139.00; *p* = <0.001) for the fourth factor of the SRAS-R. Besides, *R*^2^ de Nagelkerke ranged between 0.07 (Factor III) and 0.31 (Factor IV).

**Table 5:** Logistic regression model for the probability of presenting high school refusal behavior depending on the social functioning.

SRAS-R	CASAFS		*χ*^2^	*R*^2^	*B*	ET	Wald	*p*	OR	CI 95%
I SRAS-R	Correctly classified: 66.9%		117.01	0.19						
	SP				−0.19	0.03	45.43	<0.001	0.82	0.77–0.87
	FR				−0.15	0.03	22.36	<0.001	0.86	0.81–0.92
		Constant			5.18	0.55	88.31	<0.001	178.42	
II SRAS-R	Correctly classified: 64.9%		121.57	0.22						
	SP				−0.15	0.03	24.86	<0.001	0.86	0.81–0.91
	PR				−0.11	0.03	13.13	<0.001	0.90	0.85–0.95
	FR				−0.13	0.04	14.19	<0.001	0.88	0.82–0.94
		Constant			5.78	0.60	91.95	<0.001	326.34	
III SRAS-R	Correctly classified: 63%		30.58	0.07						
	SP				−0.11	0.03	16.12	<0.001	0.89	0.84–0.94
	FR				−0.06	0.03	4.65	0.031	0.94	0.89–0.99
		Constant			2.99	0.52	32.85	<0.001	19.86	
IV SRAS-R	Correctly classified: 78.6%		139.00	0.31						
	SP				0.26	0.05	31.94	<0.001	1.29	1.18–1.41
	PR				0.29	0.04	50.14	<0.001	1.33	1.23–1.45
	FR				−0.11	0.06	4.19	0.041	0.89	0.80–0.99
	HD				0.07	0.03	6.89	0.009	1.07	1.01–1.39
		Constant			−6.22	0.81	58.28	<0.001	0.01	

*SP, school performance; PR, peer relationships; FR, family relationships; HD, home duties/self-care; SRAS-R, school refusal assessment scale-revised; I SRAS-R, avoidance of stimuli that provoke negative affectivity; II SRAS-R, escape from aversive social and/or evaluative situations; III SRAS-R, pursuit of attention from significant others; IV SRAS-R, pursuit of tangible reinforcement outside of school*.

The value of the OR revealed that two dimensions of social functioning (school performance and family relationships) acted as negative predictors of high scores in school refusal behavior for the factors I, II, and III of the SRAS-R. Similarly, peer relationship also acted as a negative and statistically significant predictor of high scores in school refusal behavior but only for the second factor of the SRAS-R.

With regard to the fourth subscale of school refusal behavior, the dimensions of school performance, peer relationships, and home duties/self-care acted as positive and statistically significant predictors of high scores for this factor. Thus, for each point that the scores increased in those dimensions the probability of presenting high school refusal behavior based on pursuing tangible reinforcements outside of school was increased. In contrast, the family relationship dimension acted as a negative and significant predictor of high scores based on the fourth factor of the SRAS-R with a value for the OR of 0.89.

### Discussion

In the second study, the aim was to determine the role of social functioning as a protective factor of school refusal behavior. Specifically, we examined the differences in the mean scores of students with high and low school refusal behavior depending on the social functioning and analyzed the predictive capability of the social functioning on high scores in school refusal behavior.

The results found supported the hypotheses formulated for the first three factors of SRAS-R, finding that socially skillful behavior acts as a protective factor of school refusal behavior. However, the evidence found did not support the initial hypotheses for the fourth factor.

On the one hand, those students who experience negative emotions and affectivity (anxiety, social anxiety, separation anxiety or fear of negative evaluation) to the obligation to attend school have reported lower scores on social functioning. Specifically, those students with high scores in school refusal behavior based on the first three factors of SRAS-R, which are associated with feelings of negative affectivity, social anxiety, evaluation worries, and pursuing attention, showed low scores in the following dimensions of social functioning: school performance, peer relationships, and family relationships. These results are in accordance with the fifth hypothesis. In turn, these dimensions of social functioning acted as negative predictors of high scores in school refusal behavior, confirming the sixth hypothesis. These findings are in line with results by Gonzálvez et al. (2019), who identified that school refusers by mixed reinforcement profile, characterized by high scores in the first three factors of the SRAS-R, scored the lowest scores on social functioning in comparison with the rest of profiles. It is common for students with this type of school refusal to experience difficulties in social interaction, expressing poor interpersonal skills, and avoiding aversive social situations or evaluations (Egger et al., 2003; Kearney and Albano, 2004; Jones and Suveg, 2015). In these cases, the acquisition of skills that promote effective social behavior would act as a positive factor against school refusal.

On the other hand, children who refuse to attend school because of pursuing tangible reinforcements outside the school (Factor IV) got opposite results. In this case, students with high school refusal behavior scored higher on all the dimensions of social functioning. Besides, logistic analyses revealed that three of the dimensions that formed part of the social functioning construct (school performance, peer relationships, and home duties/self-care) acted as positive predictors of high scores in school refusal behavior. This result is also consistent with Gonzálvez et al. (2019) findings in which the school refusal behavior profile with high scores in the fourth factor of the SRAS-R obtained together with the non-refusal group the highest scores in social functioning. However, having not found more previous investigations in this field, it is necessary to expand the research in order to check which perception of reality these students show and contrast it through multi-source studies including teachers and relatives. With this type of works, responses based on thoughts that do not fit with the real context could be detected and alternative explanations could be proposed (Holmbeck et al., 2002). On the contrary, the probability of presenting high scores in school refusal for the fourth factor was lower as scores increased in family relationships. In this case, the consolidation of a favorable relationship with family members would act as a protective factor of school refusal, coinciding with those investigations that highlight the protective influence exerted by an adequate family context (parenting style, family structure, and climate) on school refusal (Bahali et al., 2011; Carless et al., 2015).

## Conclusions

Scientific literature review indicates the need to validate specific instruments that evaluate the adaptive social functioning in new cultural contexts (Price et al., 2002). In this sense, the present investigation offers the first Spanish validation of the CASAFS and demonstrates the solvency and effectiveness of this scale to assess this variable in Spanish children population. Moreover, this study is framed within a new perspective that seeks to identify which factors act as protectors of school refusal behavior. The negative to attend school has been commonly associated with internalizing problems such as anxiety or depression (Heyne et al., 2011; Richards and Hadwin, 2011; Gonzálvez et al., 2018a,b), externalizing problems such as disruptive behaviors (Egger et al., 2003; Maynard et al., 2012) and low academic performance (Barry et al., 2010; Yahaya et al., 2010; Thornton et al., 2013). From these data, the interest in detecting those variables that negatively affect students who refuse or show difficulties to attend school is evident. However, this study pretended to overcome this view by offering the first results about the role of social functioning as a protective factor of school refusal behavior.

Despite these findings, this study has some limitations that must be mentioned. First, for achieving a more exhaustive validation of the CASAFS, additional studies are required to verify the temporal stability of this measure and the convergence validity between this scale and other similar instruments. Second, the little scientific research evaluating the relationship between social functioning and school refusal behavior does not allow generalizing these findings. Therefore, it is necessary that future studies expand the study of the relationship between these variables in order to provide greater consistency and validity to the results found. In addition, this study only analyzes information provided by students through two self-report measures. In order to avoid responses conditioned by the subjective view of the students, it is proposed that future works include opinions of teachers and parents as well as the use of different evaluation tools (e.g., interviews or observational instruments). On the other hand, although the number of participants constitutes a representative sample of the stage of primary education, it is not possible to generalize the results obtained to other ages. Therefore, it is proposed to develop future analysis in higher educational stages. Additionally, this study was carried out with students who regularly attend school. This preventive approach is useful but it would be interesting comparing these findings with students who have school attendance problems. Other relevant academic factors such as school performance and school attendance rates should be considered in future works to evaluate their impact on school refusal behavior. Finally, it would be convenient to carry out longitudinal studies that allow knowing the evolution of these results over time.

Practical implications for the health educational and psychological field are derived from the results obtained. Regarding the assessment, this study provides the first validation of the CASAFS in Spanish language. Therefore, it offers a specific instrument to evaluate the social functioning skills as a prevention mechanism because several studies have suggested that deficits in social functioning are associated with psychological problems such as anxiety, depression or conduct disorders (Alfano, 2012; Essau et al., 2012; Vuthiarpa et al., 2012; García-Fernández et al., 2017). It is important, as Price et al. (2002) indicate, that reliable and valid assessment tools for social functioning are developed. In this line, early detection of deficits in social and adaptive functioning is essential to offer the more appropriate intervention strategies. With regard to school refuser students, for those students who base their refusal to attend school on feelings of negative affectivity, it is proposed the application of programs aimed at improving the emotional regulation (e.g., FORTIUS Program, Méndez et al., 2012) and social performance in young people (e.g. PEHIA Program, Inglés, 2009). On the other hand, for those students with high scores in school refusal behavior based on pursuing external reinforcements (going out with friends, staying at home playing, etc.), it is necessary to analyze how these subjects interpret their behaviors and orient them in the control and rational knowledge of the emotions generated (Inglés et al., 2015; Vicent et al., 2016). In all cases, both feelings and emotions must be properly regulated in order to improve tolerance to frustration, avoid negative emotional states, and regulate impulsivity in order to achieve adequate social functioning (Heerdink et al., 2015; Domitrovich et al., 2017; Rapp et al., 2017).

## Data Availability

The datasets for this manuscript are not publicly available because the datasets generated for this study are available on request to the corresponding author. Requests to access the datasets should be directed to carolina.gonzalvez@ua.es.

## Ethics Statement

The study followed the ethics standards established by the Declaration of Helsinki and the research study protocol was approved by the Ethical Committee of the University of Alicante (UA-2017-09-05).

## Author Contributions

CG has participated conducting a literature review and writing this manuscript. CI has participated drafting the work and revising it critically for important intellectual content in all its phases. AM-P has participated conducting a literature review and writing this manuscript. RS has participated performing statistical analyses. MV has made substantial contributions in the design and interpretation of the data. JG-F has designed this research and participated performing statistical analyses.

### Conflict of Interest Statement

The authors declare that the research was conducted in the absence of any commercial or financial relationships that could be construed as a potential conflict of interest.

The reviewer NM declared a past collaboration with several of the authors CG, CI, RS, MV, JG-F to the handling Editor.
